# Development and Validation of an MR Signal Intensity Ratio-based Discriminant Classification Model for Differentiating Nasopharyngeal Lymphoid Hyperplasia from Nasopharyngeal Carcinoma

**DOI:** 10.2174/0115734056487364260729044644

**Published:** 2026-08-03

**Authors:** Wen Li, Ying-qi Li, Ge-hua Tong, Xue-ling Qin, Xiao-feng Zou, Wei Liang, Yi-yong Liu, Jiu-ping Liang

**Affiliations:** 1 School of Chinese Medicine, Guangdong Pharmaceutical University, Guangzhou, China; 2 Department of Radiology, Shenzhen Bao’an District Songgang People's Hospital, Shenzhen, 518105, China; 3 MR Research Collaboration, Siemens Healthineers Ltd., Shenzhen, China; 4 Department of Stomatology, Shenzhen Bao’an District Songgang People's Hospital, Shenzhen, 518105, China; 5 Department of Radiology, Rehabilitation Hospital of China National Nuclear Corporation, Changsha, 410017, China; 6 Department of Radiology, Shenzhen Baoan Hospital, Southern Medical University, Shenzhen, 518101, China

**Keywords:** Magnetic resonance image, Signal intensity, Nasopharyngeal lymphoid hyperplasia, Nasopharyngeal carcinoma, Discriminant classification model

## Abstract

**Background:**

Early-stage Nasopharyngeal Carcinoma (NPC) and Nasopharyngeal Lymphoid Hyperplasia (NPLH) share similar clinical and MRI features, complicating non-invasive diagnosis. As MRI accuracy decreases with diffuse NPC signals, objective differentiation methods are urgently needed. Therefore, this study aims to evaluate the efficiency of an MRI Signal Intensity Ratio (SIR) based discriminant classification model in distinguishing NPLH from NPC.

**Materials and Methods:**

One hundred twenty-six patients with pathologically confirmed nasopharyngeal lesions (27 with NPLH and 99 with NPC) were retrospectively enrolled. The maximum, minimum, and mean signal intensities of the lesions were measured on T2-weighted imaging (T2WI) and post-contrast T1-weighted imaging (T1WI+C). Signal Intensity Ratios (SIRs) were calculated by normalizing lesion SI to that of the longus capitis muscle. A discriminant model was then developed to differentiate NPLH from NPC.

**Results:**

Stepwise discriminant analysis identified T2WI-Max-SIR and T1WI+C-Min-SIR as the optimal predictors. The established discriminant classification model yielded an original accuracy of 95.2% and a cross-validated accuracy of 93.7% in distinguishing NPLH from NPC.

**Discussion:**

NPC exhibited a significantly lower T2WI-SIR than NPLH. Incorporating these quantitative metrics into a discriminant model provides an objective assessment, minimizing scanner inconsistencies and overcoming the limitations of subjective visual evaluation.

**Conclusion:**

Integrating T2WI-Max-SIR and T1WI+C-Min-SIR into a discriminant classification model enables accurate, objective, and non-invasive differentiation between NPLH and NPC, which potentially reduces unnecessary biopsies.

## 1 INTRODUCTION

Nasopharyngeal Carcinoma (NPC), the most prevalent malignancy of the nasopharynx, is particularly common in Southeast Asia and is closely linked to Epstein-Barr virus infection, genetic susceptibility, and environmental exposures [1-3]. Global cancer statistics indicate that NPC remains highly endemic in these regions, imposing a considerable health burden with tens of thousands of new cases each year [4, 5]. While plasma Epstein-Barr virus (EBV) DNA analysis serves as a primary non-invasive screening tool, its sensitivity can fluctuate in early-stage disease, underscoring the indispensable role of advanced imaging [6]. Nasopharyngeal lymphoid hyperplasia (NPLH) is the most frequent benign lesion of the nasopharynx in adults, characterized by nasopharyngeal lymphoid hyperplasia and inflammation. Both early-stage NPC and NPLH can present with local soft-tissue thickening, nasal congestion, hearing loss, and epistaxis, making it difficult for distinguish between the two. Early diagnosis and treatment are crucial for improving the survival rate of NPC patients [7, 8]. Recent advances in fast, non-invasive screening MRI protocols have demonstrated high efficiency in the early detection of nasopharyngeal malignancies [9, 10]. Endoscopic biopsy is the gold standard for the diagnosis of NPC, but it may cause false negative results [11]. For example, it may lead to misdiagnosis when the tumor is located in the lateral recess of the nasopharynx or the submucosa, and as a result, it may require multiple biopsies or even surgery to confirm the diagnosis. In addition, nasopharyngeal biopsy is invasive and increases the risk of bleeding. Distinguishing NPLH from NPC is a significant clinical challenge due to their overlapping morphological features on conventional MRI [12]. Therefore, there is an urgent need for non-invasive methods to distinguish NPC from NPLH.

A thorough understanding of the normal nasopharyngeal anatomy and its common variations is the prerequisite for identifying pathological hyperplasia. As the best imaging modality for the diagnosis of NPC, MRI reflects anatomic information both within the lesion and of its adjacent structures [13]. MRI signs such as asymmetric thickening, heterogeneous enhancement of the nasopharyngeal wall, and cervical lymph node enlargement have high accuracy in the diagnosis of nasopharyngeal malignancies [8]. However, the diagnostic accuracy of MRI is lower when the NPC exhibits diffuse or symmetrical central signal that resembles Benign Hyperplasia (BH). In the artifact-scarce head and neck region, T2-weighted imaging (T2WI) is a standard, widely utilized sequence that demonstrates superior clinical utility over T1, T1 ratio, PD, and T2 ratio for differentiating Nasopharyngeal Carcinoma (NPC) from Benign Hyperplasia (BH) [14]. Wong *et al*. [15] used a convolutional neural network based on T2WI to achieve high sensitivity, specificity, and accuracy in the differential diagnosis of T1-stage NPC and NPLH. In addition, Contrast-enhanced T1-weighted imaging (T1WI+C) has high sensitivity for diagnosing NPC and is clinically important for differential diagnosis of NPLH and T1-stage NPC [12]. However, the signal intensity of magnetic resonance is easily affected by the magnetic field strength, Time of Echo (TE), Time of Repetition (TR), and other parameters, while the quantitative method of MR Signal Intensity Ratio (SIR) is simple and objective. Quantitative MRI parameters provide a more objective basis for the differential diagnosis of nasopharyngeal lesions. In our study, we selected the Max-SIR and Min-SIR derived from T2WI and T1WI+C sequences as independent factors. This quantitative analysis using signal intensity ratios provides a standardized approach to minimize inter-patient variability, which is more objective than traditional visual inspection.

Previous studies employing quantitative MRI SIR for differentiating nasopharyngeal carcinoma from benign hyperplasia have reported favorable diagnostic performance, supporting the use of standardized signal intensity measurements as quantitative imaging biomarkers [8]. In addition, Zhang *et al*. [14] demonstrated that the T1 ratio and T2 ratio values of T1-stage NPC were significantly lower than those in BH. Using these ratios to distinguish between stage T1 NPC and BH showed good diagnostic efficacy.

Classification and discriminant analysis are multivariate statistical technique that develops a discriminant classification model to classify new samples based on a set of known training samples and their associated variable values, according to specific discriminant criteria. This method can be applied to clinical differential diagnosis. For example, discriminant analysis is often used for two-class discrimination, which develops a discriminant classification model to objectively categorize cases based on relevant predictive variables [16]. It is objective and accurate in the diagnosis of diseases, which can avoid the influence of doctors' personal experience, and is of great significance for clinicians' diagnosis and treatment decisions. This study aimed to evaluate whether an MR-SIR-based discriminant classification model could improve diagnostic accuracy in differentiating NPLH from early-stage NPC.

## 2 MATERIALS AND METHODS

### 2.1 Patients

This retrospective study was approved by the Ethics Committee of Shenzhen Bao’an District Songgang People's Hospital, China, and individual informed consent was waived (IRB-YJ-2023-035). Analysis was conducted on 241 patients who presented with nasopharyngeal lesions identified through nasopharyngoscopy and subsequently confirmed *via* nasopharyngeal MRI. Pathological confirmation was achieved through either biopsy or surgical resection between July 2017 and July 2023. The patients sought medical attention for symptoms such as nasal congestion, epistaxis, and cervical masses. The cohort included 202 cases of NPC and 39 cases of benign nasopharyngeal lesions. Two cases of nasopharyngeal polyp, 1 case of nasopharyngeal tuberculosis, 9 cases of benign nasopharyngeal lymphoid hyperplasia, and 103 cases of NPC were excluded (Fig. **1**). A total of 126 cases were included, including 27 cases of NPLH (2 cases were treated by surgically removal, 25 cases were followed for 12 months after needle biopsy) and 99 cases of NPC. There were 82 males and 44 females. The mean age was 41.71±10.12 years, and the median age was 41 years. The inclusion criteria were as follows: (1) Nasopharyngeal lesions were identified through nasopharyngoscopy, with definitive pathological confirmation; (2) Pre-biopsy and pre-treatment nasopharyngeal MRI scans, both unenhanced and enhanced, included comprehensive imaging sequences, clear visualizations, and no significant artifacts; (3) The thickness of the nasopharyngeal lesion was ≥5mm to ensure accurate measurement; and (4) Patients with NPC were included only if at least one longus capitis muscle remained free of tumor invasion. Exclusion criteria comprised of: (1) Patients with other specific benign or infectious lesions of the nasopharynx confirmed by pathology, such as nasopharyngeal polyps or nasopharyngeal tuberculosis; (2) Patients with incomplete baseline nasopharyngeal MRI sequences, poor image quality, or severe artifacts that precluded accurate measurement; (3) Patients with a nasopharyngeal lesion thickness of < 5 mm; and (4) Bilateral longus capitis muscle invasion. The pathological types of NPC were non-keratinizing undifferentiated carcinoma. According to the Union for International Cancer Control (UICC) staging system, there were 19 cases in stage T1, 43 cases in stage T2, 27 cases in stage T3, and 10 cases in stage T4.

### 2.2 MRI Examination

Siemens Verio 3.0T and Skyra 3.0T MR Scanners were used with a head and neck coil. The scanning range was from the middle of the temporal lobe to the level of the thoracic entrance. Coronal slices were parallel to the level of the anterior edge of the C3 vertebra, and axial slices were perpendicular to the C3 vertebra. Scans included T1WI, T1WI+C and T2WI. (Table **1**). Gadobenatedimeglumine was administered at a dose of 0.1 mmol/kg and a flow rate of 3 ml/s (Spectris Solaris MR Injector System, Medrad). The axial, coronal, and sagittal images of the nasopharynx were acquired in that order, beginning 30 seconds after intravenous injection of the contrast agent through the cubital vein.

### 2.3 Image Analysis

Two radiologists with 23 years of experience in head and neck imaging measured the signal intensity of nasopharyngeal lesions and the longus capitis muscle on T2WI and contrast-enhanced T1WI+C images (RadiAnt DICOM Viewer software). Signal from the longus capitis muscle was chosen as the reference for calculating the signal intensity ratio. This choice was based on the fact that the longus capitis muscle is located deep in the neck and is less susceptible to individual variations in muscle fiber density due to chewing habits.

The Maximum Signal Intensity (Max-SI), Minimum Signal Intensity (Min-SI), and Mean Signal Intensity (Mean-SI) of the nasopharyngeal lesions were recorded. Additionally, the lesion's SIR relative to the longus capitis muscle's mean signal intensity was calculated, including Max-SIR, Min-SIR, and Mean-SIR. The Region of Interest (ROI) was defined as a round or oval area of 0.2~0.5 cm^2^ and was manually placed within the solid portion of the lesion. Areas suspected of cystic degeneration or necrosis, identified as regions with markedly high signal intensity and heterogeneous internal texture on T2WI and T1WI+C, were excluded from ROI placement. Regions affected by visible imaging artifacts were also avoided (Fig. **2**).

Signal intensity ratios were calculated according to the following formula: T1WI+C ratio = (T1WI+C mean value of lesion) / (T1WI+C mean value of longus capitis); T2WI ratio = (T2WI value of lesion) / (T2WI mean value of longus capitis).

### 2.4 Statistical Analysis

Statistical analyses were performed using SPSS version 21.0 (SPSS Inc., Chicago, IL, USA). All data were summarized as mean ± standard deviation. Intraclass Correlation Coefficients (ICCs) and a two-way random effects model were used to evaluate the consistency of the two observations (ICCs< 0.50, 0.50 to 0.75, 0.75 to 0.90, > 0.90 were considered as poor, moderate, good, and excellent consistency). The Shapiro-Wilk test was used to check the data distribution. The rank-sum test or two independent-sample *t* tests were selected for comparison. *P* < 0.05 was considered statistically significant.

Stepwise Discriminant Analysis (SDA) was applied to identify imaging biomarkers that significantly contribute to the differentiation of nasopharyngeal lesions. This method automatically selects variables through either forward inclusion (adding variables sequentially based on their significance) or backward elimination (removing non-significant variables stepwise), ultimately retaining those with the highest explanatory power for intergroup differences to be integrated into the discriminant classification model.

Discriminant analysis [17] is a multivariate statistical technique frequently used for two-class classification. It establishes the mathematical relationship between predictive variables and predefined groups and generates classification equations that can be used to assign unknown cases to the most probable category based on their characteristic profiles. This approach provides a more objective and evidence-based framework for clinical diagnosis and decision-making.

SPSS statistical package was used to construct the discriminant classification model (Fig. **3**).

### 2.5 Clinical Validation and Comparison

To comprehensively assess the robustness and clinical utility of our model, we employed two additional validation strategies beyond the original and cross-validation. First, we conducted a head-to-head comparison between our quantitative model and visual assessments in a double-blind manner by two radiologists (Doctor 1 ≤10 years of experience; Doctor 2 with >10 years of experience). Sensitivity, specificity, and balanced accuracy were computed for both observers and the model to benchmark performance. Second, to address potential selection bias and sample size imbalance, we performed a 1:1 Propensity Score Matching (PSM) analysis [18] based on age and sex, creating a balanced validation cohort (27 NPC *vs*. 27 NPLH cases). The model’s diagnostic performance was re-evaluated on this balanced dataset to ensure that the findings were not artifacts of the original class disparity.

## 3 RESULTS

### 3.1 Data Reliability and Distribution

Despite the unequal group sizes (N_1_=99, N_2_=27), a retrospective power analysis (PASS 21.0.3) demonstrated an actual power of 95.37% based on the observed effect size (α= 0.05), ensuring the statistical adequacy of the study objectives.

The ICCs between the two readers for the SIRs of lesions to the longus capitis on T2WI and T1WI+C sequences were 0.93 (95%CI: 0.92-0.94) and 0.91 (95%CI: 0.89-0.92), respectively, which suggests excellent reliability of the results provided by the two readers.

T2WI-Max-SIR, T2WI-Min-SIR, T2WI-Mean-SIR, T1WI+C -Max-SIR, T1WI+C-Min-SIR, and T1WI+C -Mean-SIR (W, *P*-values of 0.194; 0.694; 0.773; 0.289; 0.108, and 0.281) followed a normal distribution; therefore, two independent-sample *t* tests were selected for comparison.

### 3.2 Comparison of SIRs between NPLH and NPC

T2WI-Max-SIR, T2WI-Min-SIR, T2WI-Mean-SIR, and T1WI+ C-Min-SIR of NPLH and NPC were significantly different between the two groups (*P* < 0.01). There was no significant difference in T1WI+C-Max-SIR and T1WI+ C-Mean-SIR. (Table **2**).

### 3.3 Construction of the Discriminant Classification Model

(1) Utilizing a stepwise approach, the variables T2WI-Max-SIR and T1WI+C-Min-SIR were found to be statistically significant in the discrimination analysis. When T2WI-Max-SIR was incorporated into the discriminant equation, the *p*-value was 0.000; when both T2WI-Max-SIR and T1WI+C-Min-SIR were included, the *p*-value remained at 0.000. The standardized canonical discriminant classification model coefficients for T2WI-Max-SIR and T1WI+C-Min-SIR were 0.987 and -0.351, respectively. This indicates that the discriminatory power of T2WI-Max-SIR is more pronounced compared to that of T1WI+C-Min-SIR.

(2) Two classification functions generated were as follows:

Y1=-29.64+6.09×T2WI-Max-SIR+17.34×T1WI+C-Min-SIR.

Y2=-43.36+9.47×T2WI-Max-SIR+14.06×T1WI+C-Min-SIR.

T2WI-Max-SIR and T1WI+C-Min-SIR were respectively substituted into the two functions. When Y1 > Y2, the case was identified as NPC. When Y1 < Y2, the case was classified as NPLH (Fig. **4**).

### 3.4 Clinical Validation and Performance Evaluation

Original results showed that 1 case of NPC was misclassified as NPLH, and 5 cases of NPLH were misclassified as NPC, with an overall coincidence rate of 95.2%. Cross-validated results showed that 2 cases of NPC were misclassified as NPLH and 6 cases of NPLH were misclassified as NPC; the overall coincidence rate was 93.7%. (Table **3**).

To establish a rigorous clinical benchmark, we conducted a head-to-head comparison between our quantitative model and the manual assessments of two radiologists. As illustrated in Fig (**5**), the model achieved an outstanding area under the receiver operating characteristic curve (AUC) of 0.974, markedly outperforming Doctor 1 (AUC: 0.707) and Doctor 2 (AUC: 0.796). Furthermore, to evaluate diagnostic efficacy in the absence of sample imbalance, the model was validated on a 1:1 PSM-balanced cohort (Table **4**). In this balanced setting, the model retained robust performance, yielding a Balanced Accuracy of 90.7% and a Matthews Correlation Coefficient (MCC) of 0.815, values highly congruent with those observed in the original cohort (Balanced Accuracy: 90.3%, MCC: 0.855). These multi-dimensional diagnostic metrics are systematically summarized in Table **5**.

## 4 DISCUSSION

### 4.1 Diagnostic Value of MRI SIR Parameters in NPC

MRI and endoscopy are the most common methods for detecting nasopharyngeal lesions. These two methods have complementary roles in the detection of NPC [9]. MRI is more sensitive than endoscopy in detecting occult NPCs at an earlier stage; it can detect subclinical cancers that may be missed by endoscopy or biopsy; it can also effectively differentiate non-nasopharyngeal carcinoma cases, thereby reducing invasive sampling [19].

The selection of optimal MRI parameters is essential for distinguishing malignant from benign nasopharyngeal tissues. Previous studies have demonstrated that MRI, particularly with contrast enhancement, can precisely localize suspected lesions and improve guidance for biopsy, thereby enhancing diagnostic accuracy [13]. Our results showed that T1WI+ C-Min-SIR of NPC was higher than that of NPLH, which is consistent with previous reports on contrast-enhanced MRI signal behavior in nasopharyngeal carcinoma. Additionally, the T2WI-max SIR, T2WI-min SIR, and T2WI-mean SIR of NPLH were significantly higher than those of NPC, which is consistent with previous quantitative MRI studies showing that malignant lesions exhibit lower T2 signal intensity due to increased cellular density and altered tissue microstructure [20].

The difference in T2 signal intensity between NPC and NPLH may reflect not only variations in cellular density but also differences in pathological tissue microstructure and microenvironmental characteristics [21, 22]. NPC is an epithelial malignancy characterized by a high nuclear-to-cytoplasmic ratio, rapid cellular proliferation, and a tumor microenvironment with marked stromal remodeling and extracellular matrix alteration [23, 24]. Histologically, NPLH is characterized by reactive lymphoid follicles, loose stroma, and abundant interstitial fluid [25]. These structural differences dictate distinct water distribution properties: while NPC's high cellularity restricts water mobility and shortens T2 relaxation, NPLH’s loose architecture and interstitial fluid enhance water freedom, resulting in higher T2 signal intensity [8, 14].

The model consistently demonstrated superior performance across all multi-dimensional metrics compared to human experts. Specifically, on the original cohort, the model achieved a balanced accuracy of 90.3% and a cross-validation accuracy of 87.9%, significantly outperforming Doctor 1 (Balanced Accuracy: 70.7%) and Doctor 2 (Balanced Accuracy: 79.6%). Furthermore, to account for class imbalance and potential selection bias, the model was re-validated on a 1:1 PSM-balanced cohort (N=54). It maintained a robust performance with a balanced accuracy of 90.7% and an MCC of 0.815, while exhibiting stable sensitivity and specificity across all validation phases. This consistently high performance confirms the model's reliability despite initial disparities and validates its clinical potential to effectively capture morphological differences between NPLH and NPC.

### 4.2 Study Limitations

This study has several limitations that must be acknowledged. First, the lack of an independent external validation cohort necessitates caution regarding the generalizability of the model, and future multi-center studies are required for further validation. Second, Diffusion-weighted imaging (DWI) and Dynamic contrast-enhanced imaging (DCE) sequences were not included in this study; future research will investigate the potential value of these functional imaging sequences. Third, to maintain methodological homogeneity, our cohort was restricted to patients with non-keratinizing NPC, which limits the direct applicability of the model to other rare pathological subtypes. Expanding the study to include more diverse populations and different pathological subtypes will be the focus of our future research.

## CONCLUSION

This study developed a discriminant classification model using T2WI-Max-SIR and T1WI+C-Min-SIR, which enables accurate and non-invasive differentiation between NPLH and NPC. This practical quantitative method requires no advanced MRI sequences, serving as a valuable auxiliary tool for clinical diagnosis. It mitigates the limitations of invasive biopsy and subjective qualitative MRI, and offers a novel quantitative imaging approach for the early differential diagnosis of nasopharyngeal lesions.

## Figures and Tables

**Fig. (1):**
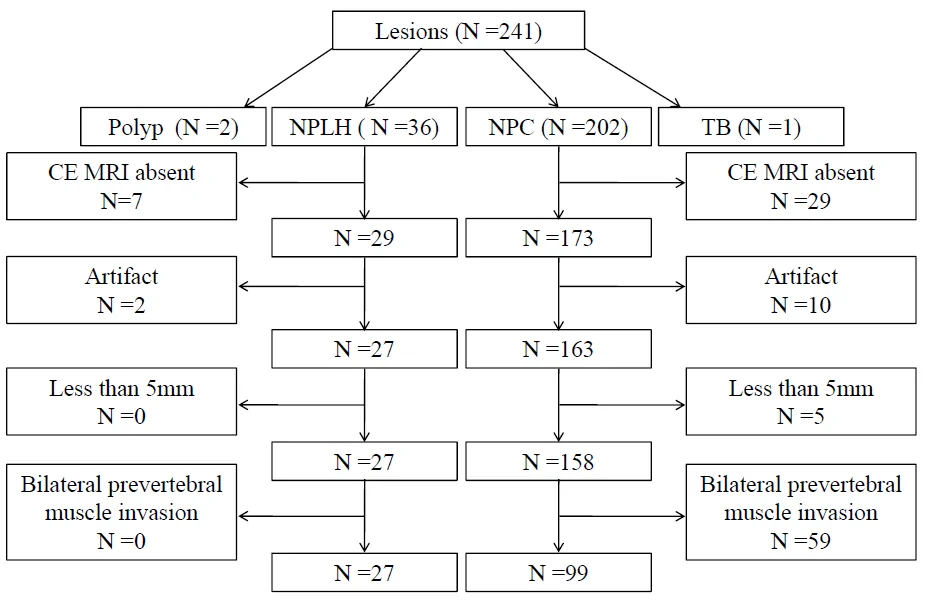
Flow diagram showing the selection of the study population.

**Fig. (2):**
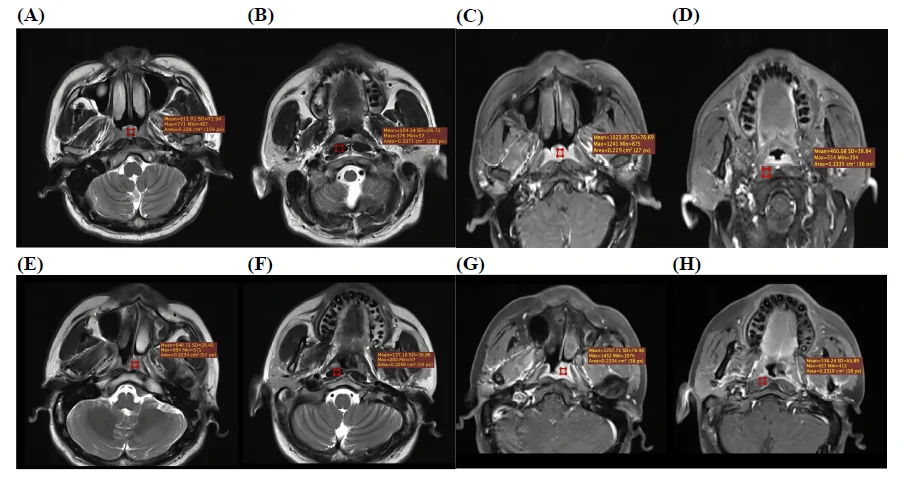
Representative case of a 38-year-old man with NPLH (A~D). The Max-SIRs, Min-SIRs, and Mean-SIRs on T2WI (**A**, **B**) and T1WI+C (**C**, **D**) images were 7.23, 4.22, 5.36, and 2.47, 1.69, 2.09, respectively. Representative case of a 33-year-old man with NPC (E~H). The Max-SIRs, Min-SIRs, and Mean-SIRs on T2WI (**E**, **F**) and T1WI+C (**G**, **H**) images were 5.42, 4.48, 4.95, and 2.57, 2.10, 2.39, respectively.

**Fig. (3):**
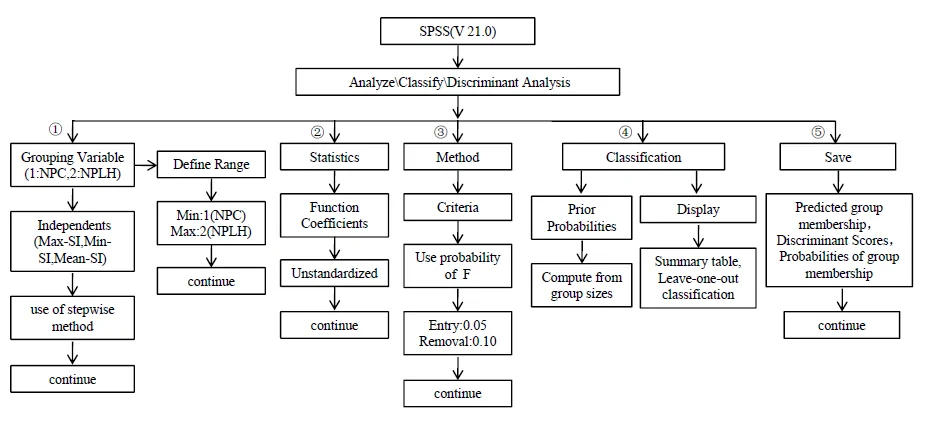
Key variables were screened through stepwise discriminant analysis by SPSS and used to construct the discriminant classification model.

**Fig. (4):**
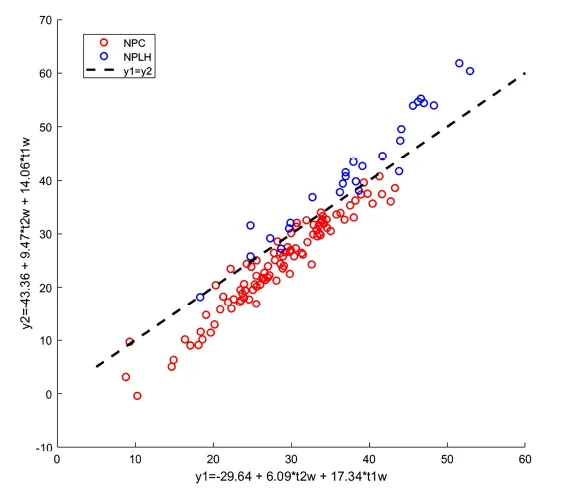
Two classification functions were generated. When Y1 > Y2, the case was identified as NPC. When Y1 < Y2, the case was classified as NPLH.

**Fig. (5):**
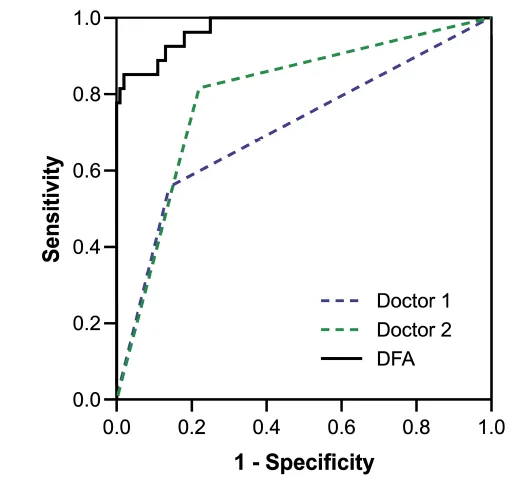
As shown by the ROC curve, the Discriminant Function Analysis (DFA) model achieved superior diagnostic performance (AUC = 0.974) relative to the human experts (AUC = 0.707 and 0.796). This suggests the model offers substantial value as an adjunct to conventional visual assessment for distinguishing NPC from NPLH.

**Table 1:** Magnetic resonance imaging sequence parameters.

Sequence	TE(ms)	TR(ms)	Thickness/spacing(mm)	FOV(mm)	Matrix	NEX
Tra T1WI	9	639	3/0.6	230×230	256×256	1
Tra T2WI	92	6120	3/0.6	230×230	256×256	1
Tra T2WI-FS	92	6120	3/0.6	230×230	256×256	1
Cor T2WI-FS	82	6000	4/0.8	300×300	320×224	1
Sag T2WI-FS	82	6000	4/0.8	300×300	320×224	1
Tra T1WI+C	9	639	3/0.6	230×230	256×256	1
Cor T1WI+C	9.5	750	4/0.4	300×300	320×224	1
Sag T1WI+C	9.5	750	3/0.3	300×300	320×224	1

**Abbreviations:** TE, echo time; TR, repetition time; FOV, field of view; NEX, number of excitations; FS, fat suppression; Tra, Transverse; Cor,Coronal; Sag, Sagittal.

**Table 2:** The difference in signal intensity ratio between NPLH and NPC.

	T2WI			T1WI+C		
	Max-SIR	Min-SIR	Mean-SIR	Max-SIR	Min-SIR	Mean-SIR
NPLH	6.70±0.99	4.40±0.65	5.49±0.71	2.25±0.39	1.55±0.30	1.89±0.30
NPC	4.59±0.76	3.04±0.73	3.83±0.69	2.22±0.36	1.73±0.29	1.97±0.28
*t*	12.02	8.76	11.09	0.42	2.66	1.16
*P*	0.000	0.000	0.000	0.403	0.011	0.252

**Table 3:** Classification results of original and cross-validated.

Group	Original		Cross-validated		Total
	NPLH	NPC	NPLH	NPC	
NPC	1(1%)	98(99%)	2(2%)	97(98%)	99
NPLH	22(81.5%)	5(18.5%)	21(77.8%)	6(22.2%)	27

**Table 4:** For the 1:1 PSM-balanced cohort (N=54).

Group	Original		Cross-validated		Total
	NPLH	NPC	NPLH	NPC	
NPC	2(7.4%)	25(92.6%)	2(7.4%)	25(92.6%)	27
NPLH	24(88.9%)	3(11.1%)	24(88.9%)	3(11.1%)	27

**Table 5:** Comparison of discriminant model performance metrics before and after propensity score matching (PSM).

	Sensitivity (NPC %)	Specificity (NPLH %)	PPV (%)	NPV (%)	Balanced Accuracy	MCC
Original (N=126)	99	81.5	95.1	95.7	90.3	0.855
Cross-validated (N=126)	98	77.8	94.2	91.3	87.9	0.805
Doctor 1(N=126)	85.9	55.6	87.6	51.7	70.7	0.404
Doctor 2(N=126)	77.8	81.5	93.9	50.0	79.6	0.510
PSM(N=54)	92.6	88.9	89.3	92.3	90.7	0.815

## Data Availability

The data and supportive information is available within the article.

## LIST OF ABBREVIATIONS

NPLH: Nasopharyngeal lymphoid hyperplasia
MRI: Magnetic resonance imaging
T1WI: T1-Weighted image
T2WI: T2-Weighted image
TR: Time of repetition
TE: Time of echo
SIR: Signal intensity ratio
BH: Benign hyperplasia
ROI: Region of interest
Tra: Transverse
Cor: Coronal
Sag: Sagittal
DWI: Diffusion-weighted imaging
DCE: Dynamic contrast-enhanced imaging

## STANDARDS OF REPORTING

STROBE guidelines were followed.
